# The importance of the urologist in male oncology fertility preservation

**DOI:** 10.1111/bju.15772

**Published:** 2022-05-31

**Authors:** Lionel A. Micol, Funmi Adenubi, Elizabeth Williamson, Sheila Lane, Rod T. Mitchell, Philippa Sangster

**Affiliations:** ^1^ Institute of Andrology University College London Hospitals NHS Foundation Trust London UK; ^2^ Urology CHUV Lausanne Switzerland; ^3^ CPMA Lausanne Switzerland; ^4^ Reproductive Medicine Unit University College London Hospitals NHS Foundation Trust London UK; ^5^ Children's Haematology and Oncology Oxford University Hospitals NHS Foundation Trust Oxford UK; ^6^ Centre for Reproductive Health Edinburgh Royal Hospital for Sick Children The University of Edinburgh MRC Edinburgh UK

**Keywords:** fertility preservation, cancer, chemotherapy, azoospermia, cryptozoospermia, surgical sperm retrieval

## Abstract

**Objectives:**

To demonstrate that surgical sperm retrieval (SSR) and spermatogonial stem cell retrieval (SSCR) in an oncological context are safe and successful.

**Patients and Methods:**

This a retrospective study in a tertiary hospital in the UK. Patients requiring fertility preservation from December 2017 to January 2020 were included. Data were analysed with Microsoft Excel 2016 and the Statistical Package for the Social Sciences (version 20).

**Results:**

Among 1264 patients referred to the Reproductive Medical Unit at the University College of London Hospitals for cryopreservation prior to gonadotoxic treatment, 39 chose to go forward with SSR/SSCR because they presented as azoo‐/cryptozoospermic or an inability to masturbate/ejaculate. Interventions were testicular sperm extraction (23 patients) or aspiration (one), electroejaculation (one), and testicular wedge biopsy for SSCR (14). The median (range) age was 15.0 (10–65) years and the median testosterone level was 4.4 nmoL/L. Primary diagnoses were sarcoma in 11 patients, leukaemia in nine, lymphoma in eight, testicular tumour in five, other oncological haematological entities in two, other solid cancers in two, while two patients had non‐oncological haematological diseases. SSR/SSCR could be offered within 7.5 days on average. Chemotherapy could follow within 2 days from SSR/SSCR, and bone marrow transplant occurred within 19.5 days (all expressed as medians). The success rate for SSR was 68.0% (at least one vial/straw collected). The mean (SD) Johnsen score of testicular biopsies was 5.23 (2.25) with a trend towards positive correlation with SSR success (*P* = 0.07). However, age, hormonal profile and type of cancer did not predict SSR outcome.

**Conclusion:**

We show that SSR and SSCR in an oncological context are valid treatment options with a high success rate for patients in which sperm cryopreservation from semen is impossible. By providing an effective pathway, fertility preservation is possible with minimal delay to oncological treatment.

## Introduction

Fertility preservation (FP) in men and boys is increasingly recognised as a vital part of oncological treatment, with guidelines now recommending that FP is discussed with all patients about to undergo cancer or medical treatment that may affect their fertility [1, 2, 3].

Currently, the only established clinical option for FP in these male patients is cryopreservation of sperm after masturbation. However, it is not always possible to obtain a semen specimen as a result of physical, psychological, or cultural factors or azoospermia at presentation.

Traditionally, oncologists or haematologists have undertaken these initial discussions and organised for sperm cryopreservation, where deemed appropriate. However, if the patient is unable to ejaculate or found to be azoospermic this is frequently the end of the line for FP. There is often a concern that cancer treatment must be started as soon as possible, and any form of surgical sperm retrieval (SSR) will result in unacceptable delays. However, this does need to be weighed up with the excellent long‐term outcomes particularly for paediatric cancers (10‐year survival rates of 76% in the UK for all childhood cancer diagnosed in Great Britain between 2001 and 2005 and 5‐year survival rate of 82% but similar 10‐year survival between 2006 and 2010 [4, 5]).

Men and adolescents who are at risk of future infertility but unable to produce a semen sample can be referred to a urologist for investigation and consideration of urgent SSR. Formal pathways and a fast referral service are uniquely offered at our hospital, whereby we aim to review any oncology patient within 24 h of referral and if needed a SSR is organised, which is expedited and carried out as a matter of urgency.

This study aimed to evaluate the success rate for SSR and quantify the delay in cancer treatment for all male patients requiring FP and referred to the andrology team. In addition, the options available for younger boys who are either pre‐ or peri‐pubertal will also be discussed in this article.

## Patients and Methods

We conducted a retrospective cohort study involving patients treated at a single‐centre tertiary referral hospital, namely University College of London Hospital (UCLH), from December 2017 to January 2020. All patients who had been referred for semen cryopreservation prior to undergoing treatment with a high risk of subsequent infertility, including orchidectomy, chemotherapy and/or bone marrow transplant were included.

Azoospermic (no sperm) or cryptozoospermic (spermatozoa cannot be observed in a fresh semen sample, but potentially after extended centrifugation and following microscopic search) patients (according to WHO standards) as well as patients unable to ejaculate, either because they were too unwell or actually suffering from anejaculation (inability to orgasm) were referred to a urologist for assessment and FP counselling. After referral, an initial meeting with the patient would be organised, usually within 24 h.

Referrers were asked to organise urgent hormonal profiles (testosterone, follicle‐stimulating hormone [FSH] and luteinising hormone [LH]), which ideally should be taken first thing in the morning and with the patient fasting. Sex hormone levels can be very deranged in some of the very unwell patients and can give an indication of poor spermatogenesis thus directing potential surgical treatments.

In younger patients, clinical parameters, including Tanner stage and biochemical hormonal profile levels, as well as sensitive discussions on sexual function and masturbation, were used to assess the likelihood of spermarche and the chance of finding sperm at the time of the SSR. The Tanner stage is an objective classification system, which is a scale of physical development based on external secondary sexual characteristics, that aims to identify the stage of puberty that an adolescent is in. Biochemical hormonal levels may be useful to indicate spermatogenesis in these young patients [6].

Prepubertal boys, in whom spermatogenesis would not yet be present, can be listed for a testicular biopsy in order to take a small amount of tissue to cryopreserve immature testicular tissue containing spermatogonial stem cells (SSCs), an approach that remains experimental.

In a small number of cases where the urgency to start chemotherapy prevented FP being carried out, the collection of testicular tissue for SSC preservation between the first few treatment cycles of treatment may be considered. SSCs should still be present, and storage of the tissue will then give patients options for fertility restoration at some future time point, although it must be made clear that established clinical options for this are not yet available. Mature sperm in contrast should not be stored after commencing chemotherapy as this exposure could lead to mutagenic changes in the mature sperm [7]. Whilst mutagenic damage to the SSCs may also occur it is likely that germ cells deriving from damaged SSCs would fail to complete spermatogenesis as a result of the meiotic checkpoints that can repair damaged DNA or trigger apoptosis [8]. This is supported by data showing no increased risk of adverse pregnancy outcomes or congenital anomalies in offspring born to fathers who received chemotherapy in childhood [9, 10].

**Fig. 1:**
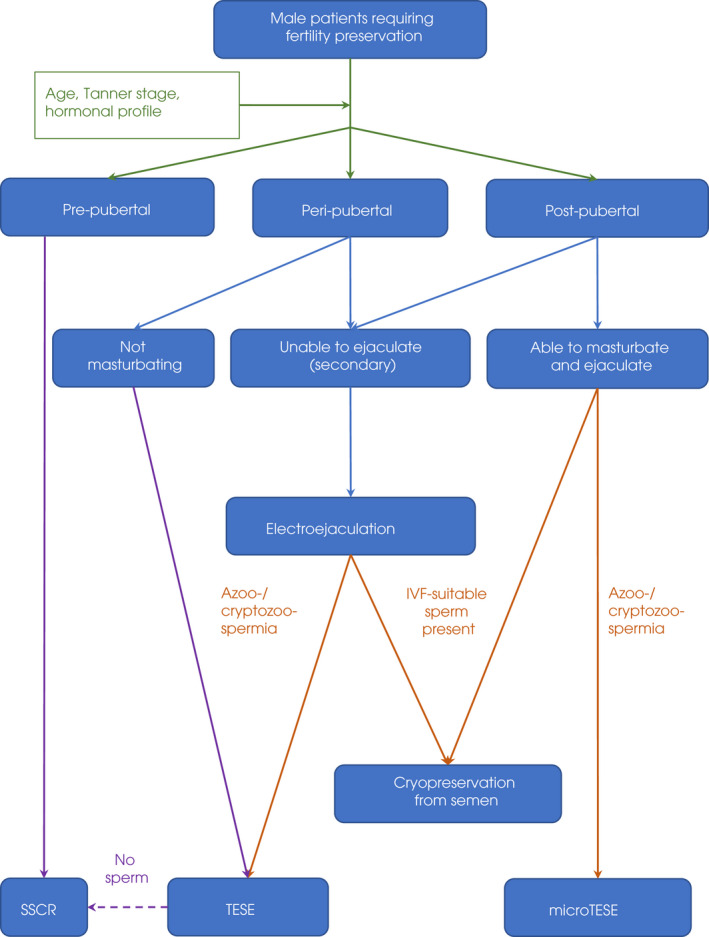
Fertility preservation flowchart. Green arrows = clinical evaluation of puberty/hormonal status; blue arrows = ejaculation abilities; beige arrows = semen examination; purple arrows = pathways towards SSCR within the frame of approved protocols (in keeping with the PanCareLife international consensus guidelines, DOI: 10.1002/cncr.30047). IVF, in vitro fertilisation.

Fertility preservation options offered included electroejaculation (EEj), percutaneous epididymal sperm aspiration (PESA), testicular sperm aspiration (TESA), testicular sperm extraction (TESE), microTESE (where an operating microscope is used to find healthy seminiferous tubules, increasing the chance of successful sperm retrieval [11]) or SSC retrieval (SSCR) via harvesting of testicular tissue for cryopreservation. A flow chart summarising patient‐matched decision for FP procedure is shown in Fig. 1.

Electroejaculation was performed under general anaesthesia for patients unable to ejaculate using the Seager Model 14 Electroejaculator (Dalzell USA medical systems). An anal electrode is inserted into the rectum and comes into contact with the region of the prostate and seminal vesicles. Electrical stimulations are administered in order to produce ejaculation as previously described [12]. An embryologist in theatre would assess the sample to decide if it was suitable for cryopreservation and if not, a TESE was performed under the same anaesthetic.

In those patients who had been able to ejaculate but found to have azoospermia, they would undergo a SSR. This usually involved a small 3‐cm incision on the scrotum and was carried out under a general anaesthetic, or local anaesthetic with sedation if the patient was considered unfit for a general anaesthetic.

The least invasive procedure possible would always be carried out but ensuring enough sperm could be cryopreserved for multiple assisted reproductive cycles in the future. The secondary aim would be to limit surgical risks of haematoma or wound issues that might delay oncological treatment.

The least invasive surgical procedure, a PESA was reserved for obstructive azoospermia cases, which is uncommon in this patient group. In cases of non‐obstructive azoospermia and cryptozoospermia, a small unilateral TESE was performed and then depending on the results relayed by the embryologists in theatre, a decision would be made on the necessity of using the operating microscope and carrying out a microTESE and if a bilateral testicle procedure was needed. Whatever the decision, this could all be carried out through the same, small scrotal incision. For testicular cancers, oncological TESE (onco‐TESE) was performed as previously described [13].

Success rate for SSR was defined as enough sperm frozen for at least one intra‐cytoplasmic sperm injection (ICSI) cycle; that is at least one stored vial or straw cryopreserved.

Boys who were clearly prepubertal may be offered SSCR by testicular biopsy and storage to preserve SSCs, although this remains experimental. The procedure involves a small scrotal incision and testicular wedge biopsy.

In peri‐pubertal cases where spermatogenesis was uncertain, both the embryologist and the Oxford Cell and Tissue Biobank (OCTB) team would be present in theatre. A small testicle biopsy would be analysed intraoperatively and depending on the findings of mature spermatozoa present under the microscope, a decision would be made on suitability for either sperm cryopreservation or SSCR.

Hospitals offering SSCR need to have an arrangement with a Biobank that holds a Human Tissue Authority (HTA) licence for the procurement, storage, and distribution of testicular tissue. The UCLH have such an agreement with the OCTB. The OCTB arrange for consent for tissue storage and attendance in theatre to collect and transport tissue to the OCTB for processing and storage. Cryopreservation of the SSC involves a controlled slow‐freezing protocol.

Data were analysed with Microsoft Excel 2016 and Statistical Package for the Social Sciences (SPSS®), version 20.0 (IBM Corp., Armonk, NY, USA). The latter was used for step‐by‐step descending multinomial logistic regression (final significance level *P* < 0.05) with inclusion following univariate multinomial logistic regression (significance level *P* < 0.20).

## Ethics

Data from UCLH patients was retrospectively collected as observations from the clinical hospital notes and do not require a separate ethical review according to the NHS Health Research Authority assessment.

Fertility preservation treatment involving storage of testicular tissue containing SSCs is performed at the Oxford University Hospitals NHS foundation Trust (OUHFT) under HTA Human Application Licence, as part of the Future Fertility Programme Oxford (FFPO). The FFPO is a clinical programme and does not require research and development and ethical approval. The programme has been approved by OUHFT Technical Advisory Group and Clinical Ethics Group. The PanCareLIFE Consortium and the International Late Effects of Childhood Cancer Guideline Harmonisation Group [14] published Ethical Guidelines for storage of reproductive tissue and the FFPO programme is fully aligned with the published guidance.

## Results

From December 2017 to January 2020 1264 patients were referred for sperm cryopreservation prior to gonadotoxic treatment, of which 1140 attended. Among those patients, 58 (5.1%) were unsuccessful: 32 (2.8%) were azoospermic, nine (0.8%) had cryptozoospermia without viable sperm and 17 (1.5%) were unable to provide a semen sample on the day. Out of the 58 eligible patients, 39 (67.2%) chose to go forward with SSR/SSCR. The median (range) age was 15.0 (10–65) years and the median testosterone level was 4.4 nmoL/L, while the overall mean (SD) Johnsen score of testicular biopsies was 5.23 (2.25). Tanner stage of puberty, cancer type, and hormone levels are described in Tables 1 and 2 for patients benefiting from SSCR and SSR, respectively.

**Table 1:** Characteristics of patients undergoing SSCR.

Age, years	Cancer type	Masturbating?	Tanner stage	FSH, IU/L	LH, IU/L	Testosterone, nmol/L
10	Ewing sarcoma	No	n/a	n/a	n/a	n/a
11	Osteosarcoma	n/a	1	1	0.7	0.1
12	Ewing sarcoma	No	1	3.1	5.7	0.8
12	Ewing sarcoma	No	2	0.3	0.3	0
13	Leukaemia	n/a	2	n/a	n/a	n/a
13	Ewing sarcoma	n/a	2	2.2	0	0.1
13	Osteosarcoma	No	2–3	1.7	2.7	1.5
13	Hodgkin lymphoma	No	2–3	0.6	0.9	0.4
14	Leukaemia	n/a	n/a	n/a	5.2	4.9
14	Ewing sarcoma	No	2	n/a	n/a	n/a
15	Leukaemia	Yes	4	n/a	n/a	n/a
15	Leukaemia	n/a	n/a	n/a	n/a	n/a
16	Leukaemia	Yes	4	4.9	15	7.6
18	Leukaemia	Yes	4	n/a	5.3	5.5

n/a, not available.

**Table 2:** Characteristics of patients undergoing SSR.

Age, years	Cancer type (or other disease)	Tanner stage	FSH, IU/L	LH, IU/L	Testosterone, nmol/L	Reason for SSR	Johnsen score
12	Ewing sarcoma	2	2.0	n/a	2.5	Failure to provide	5.8
12	Ewing sarcoma	2–3	2.4	1.8	11.1	n/a	5.5
14	Anaplastic large‐cell lymphoma	3	2.7	1.0	0.8	Cryptozoospermia	7.1
14	Medulloblastoma	3	9.5	n/a	23.0	Failure to provide	n/a
14	Testicular germ cell cancer	3	n/a	n/a	n/a	Failure to provide	n/a
14	Testicular germ cell cancer	4	13.1	9.2	9.5	Azoospermia	n/a
15	Diamond‐Blackfan anaemia	4	9.7	2.5	20.7	n/a	n/a
15	Prostate Rhabdomyosarcoma	4	3.6	2.6	7.0	Failure to provide	7.6
16	Primary immunodeficiency	4	3.3	3.7	8.4	Failure to provide	6.8
16	Leukaemia	3	12.8	n/a	5.1	Azoospermia	2
17	B‐cell lymphoma	4	4.6	3.4	7.5	Failure to provide	5.48
18	Myelodysplastic syndrome	4	8.9	11.9	25.6	n/a	5.07
18	Testicular germ cell cancer	n/a	n/a	n/a	n/a	Azoospermia	n/a
19	B‐cell lymphoma	5	4.0	4.1	n/a	Failure to provide	6.97
19	Hodgkin lymphoma	4	5.1	10.7	3.0	Cryptozoospermia	2.55
21	Rhabdomyosarcoma	5	6.0	12.3	2.9	n/a	7.4
22	Lymphoma	5	4	n/a	3.3	Failure to provide	2.25
23	B‐cell lymphoma	5	7.2	n/a	3.4	Anejaculation	9.95
23	Hodgkin lymphoma	n/a	6.0	3.8	n/a	Azoospermia	6.1
25	Burkitt lymphoma	5	2.2	n/a	0.8	Failure to provide	n/a
28	Multiple myeloma	5	2.2	5.2	3.7	n/a	n/a
29	Testicular Pick nodule	4	14.1	17.0	20.3	Cryptozoospermia	7.1
31	Testicular Leydig cell tumour	n/a	40.5	23.2	15.1	Azoospermia	n/a
32	Leukaemia	5	7.0	12.1	6.3	Cryptozoospermia	5
65	Prostate cancer	5	5.3	4.1	13.2	Anejaculation	n/a

n/a, not available.

The SSR or SSCR surgery was offered within a median (range) duration of 5.0 (0–19) days from referral. Chemotherapy was started within a median (range) of 2.0 (−115 to 41) days from the date of surgery.

Five patients underwent their stem cell harvesting after starting chemotherapy, between the first and third cycles.

When applicable, bone marrow transplant occurred within a median (range) of 19.5 (3–154) days from SSR or SSCR, while chemotherapy prior to bone marrow transplant could be started 1 or 2 days after surgery. There was no delay for chemotherapy treatment for any patient secondary to a surgical complication.

**Fig. 2:**
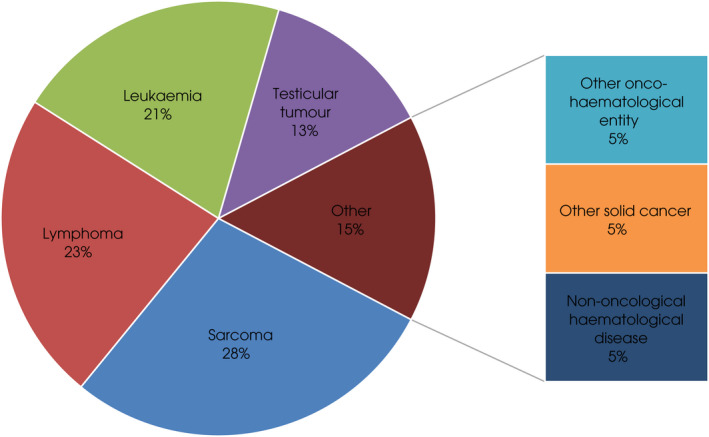
Primary diagnosis of patients.

Oncological primary diagnoses are summarised in Fig. 2 and detailed in Tables 1 and 2. The majority of patients were referred from haematology departments (53.8%).

**Fig. 3:**
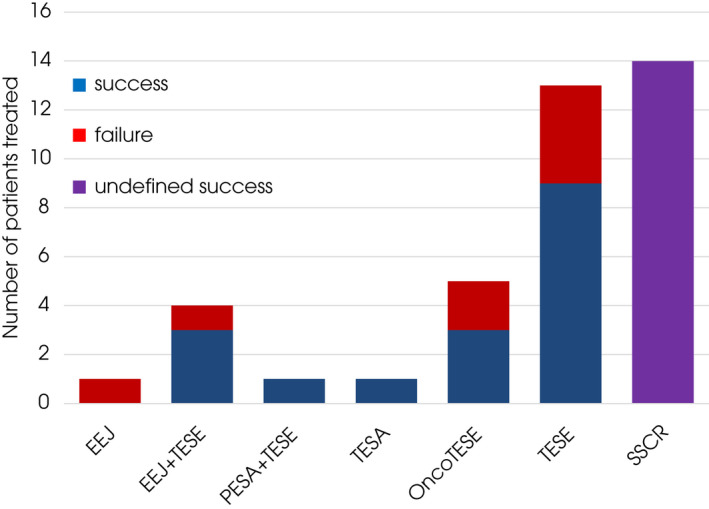
Success of SSR/SSCR according to subtype. *Further success in terms of live birth from IVF/ICSI yet unknown.

Among patients undergoing SSR, 23 patients had TESE: including microTESE (13), onco‐TESE (five), unsuccessful EEj followed by standard TESE (four), and PESA followed by standard TESE (one). Other forms of SSR were TESA (one patient) and EEj only (one). The success rate for SSR overall was 68.0% (17/25) with a mean (SD) of 5.2 (4.7) vials/straws stored. The success rates were 60.0% for EEj ± TESE, 60.0% for onco‐TESE and 69.2% for microTESE. Failed SSR comprises failed EEj and failed microTESE. In all, 14 pre‐ or peripubertal boys had testicular wedge biopsy for SSCR after failure to observe sperm intraoperatively from a single biopsy (microTESEwas not attempted in this subset of patients). The detail of both SSR and SSCR is depicted in Fig. 3.

In our data set, the success rate of SSR did not correlate with either age or hormonal levels whether LH, FSH or testosterone but numbers are small to comment on significance. As for men initially found to be azoospermic, SSR was successful in two of five compared with three of four cryptozoospermic men, two of two anejaculating patients, and seven of nine unable to provide a sample.

Moreover, the underlying oncological diagnosis was not significantly correlated with success of SSR. Mean Johnsen score as expected showed a positive trend of correlation with SSR success (*P* = 0.07).

No adverse effect from SSCR/SSR were reported among the 39 patients. Two patients died due to their advanced oncological pathology before oncological treatment but this was not related to the FP procedure.

## Discussion

Fertility preservation is recommended for patients before medical treatment that may affect their fertility. Infertility including azoospermia is more common in oncology patients prior to any gonadotoxic treatment than the general population [15]. Therefore, SSR may be necessary for these patients. The reported rate of patients with cancer able to cryopreserve sperm in this study (94.9%) is slightly higher than previous reports within the literature, showing success rates up to 89% in adults and 80% in adolescents [16, 17, 18]. Indeed, sperm quality is significantly lower in patients with cancer especially those with leukaemia or testicular tumours [19, 20, 21, 22]. Although the numbers in this study are small, the success rate of emergency SSR in an oncological context is comparable to the previously reported 64% success rate for EEj and slightly higher than the previously reported 38% success rate for TESE [17]. In addition, our success rate compares better with the 62% success rate of TESE for male‐factor infertility [23]. As for predictors of SSR success, maximum Johnsen score is known to be a better positive predictor than mean Johnsen score [24, 25], which could explain why we could only show a trend with our data, as we only had mean Johnsen score available.

All patients, for whom planned treatment of an underlying condition could potentially cause infertility, should have a consultation in which fertility and available FP treatments are discussed. The discussion must include a clear explanation of the level of infertility risk and details about the potential risk and benefits of FP treatment. For cancer treatment, the risk of infertility is high in protocols including total body irradiation, radiation to the testes, cranio‐spinal irradiation, chemotherapy conditioning regimens pre‐stem cell transplant, or treatment protocols that include high‐dose alkylating agents. Gonadotoxic risk for each of the current cancer treatment protocols has been investigated and expressed as cyclophosphamide equivalent dose [26, 27]. As the risks associated with standard sperm storage are low, all males able to store sperm should be offered the opportunity to do so before starting gonadotoxic treatment. Even when the planned treatment carries a lower risk of gonadotoxicity, obtaining sperm before any treatment gives the best chance of storing viable sperm should future relapse or escalation of treatment be required before a safe window for sperm banking can be attained. In our cohort, four (10%) patients attempted FP due to relapse, with successful sperm storage for two.

Whilst obtaining sperm from males due to undergo gonadotoxic therapies offers the potential for future fertility using assisted reproductive technologies (in general this would be an ICSI cycle), this is not possible for prepubertal boys. Currently, there are no proven clinical options for fertility preservation or restoration in this patient group [28]. Over recent years, there has been increased interest in carrying out surgery to obtain cryopreserved testicular tissues from boys before treatments that have a high risk of infertility [29]. The aim of this approach is to preserve viable SSCs within the tissue. The samples are stored as whole tissue keeping the SSCs within the SSC niche. This allows use of the stored material as whole tissue auto‐transplantation, isolated stem cell re‐infusion or in vitro sperm production. All three methods have been shown to be successful in animal models with resultant live healthy offspring but the optimal way for tissue to be used in humans is still a matter of research. [28]. To date >1000 boys worldwide have had testicular tissue cryopreserved and this includes >300 boys in the UK, in approved centres in Oxford and Edinburgh [29, 30]. Most of these boys have a cancer diagnosis, although there are an increasing proportion of boys receiving gonadotoxic therapies as conditioning for bone marrow transplantation for benign conditions [29]. As this is still considered experimental, this approach should be conducted under an HTA licence with research as a scheduled purpose for the stored tissue.

Whilst survival of SSCs has been demonstrated within frozen–thawed tissues [31], functional capacity of the SSCs in the cryopreserved patient tissues is still uncertain in humans. Options for generation of sperm from cryopreserved prepubertal testicular tissues would include in vitro spermatogenesis, SSC transplantation and testicular tissue transplantation. Each of these approaches have proven effective in animal models, although none of them have yet been translated into clinical practice [32]. Testicular tissue transplantation involves replacement of frozen–thawed testicular tissues back to the patient after their treatment is completed. This offers the potential for generation of sperm from the SSCs within the tissue. This method would not restore natural fertility as a direct connection between the seminiferous tubules of the transplanted tissue and the testis will not be restored. Therefore, sperm generated using this approach would need to be extracted from the tissue for ICSI to generate progeny. Whilst this approach has never been attempted in humans, this has been successful in several animal species including recent studies in non‐human primate models [33, 34]. These proof‐of‐principle studies have shown that this may be a viable option for patients in the future; however, caution will need to be taken to ensure that there is no risk of re‐introducing malignant cells back into the patient [35].

An alternative approach that has the added advantage of restoring natural fertility is SSC transplantation. This approach requires dissociation of the cryopreserved testicular tissue and isolation of SSCs. The SSCs can be injected directly into the seminiferous tubules after completion of treatment. Animal studies have shown that this is feasible and the SSCs re‐colonise the testis resulting in restoration of spermatogenesis and fertility (reviewed in Kilcoyne et al. [28]. An additional benefit of this approach compared with transplantation of whole tissue is that malignant cells can be excluded from the cells that are injected avoiding the potential for re‐introducing cancer to the patient. Whilst this is an attractive option for future clinical application a major limitation is the low number of SSCs present within the testis. Whilst the number of SSCs can be significantly increased by in vitro propagation in rodent models, the number of SSCs present in prepubertal human testis tissue is limited and effective methods to propagate SSCs in vitro will also be required for human testis tissues [36].

For patients in whom tissue or cells cannot be transplanted, e.g., high risk of re‐introduction of malignancy, in vitro spermatogenesis may be a future option. This involves culture of whole tissue to generate sperm from the SSCs within the tissue. Similar to the transplantation approaches, animal models have demonstrated proof‐of‐principle that this can be effective. Sperm generated from in vitro culture of neonatal rodent tissues have been used for ICSI to generate offspring [37]. Recent reports of in vitro spermatogenesis using human testis tissues have emerged [38, 39], although a full characterisation of the haploid ‘sperm‐like’ cells and demonstration of their functional potential has yet to be reported. Given that these sperm will be generated under artificial conditions, the genetic and epigenetic stability of these gametes would require careful analysis and follow‐up of any offspring generated using this approach would be imperative. In summary, while several options for fertility restoration using cryopreserved prepubertal testis tissues have shown promise in animal models, they have not yet been shown to be effective as clinical therapies in humans. Development of these approaches for clinical application will require further pre‐clinical study followed by clinical trials with a focus on safety for the patient and for subsequent generations that result from artificially‐derived gametes.

Testicular tissue cryopreservation for the storage of SSCs is still a relatively new experimental technology in the UK. To date this research activity is only partially funded by the NHS but work is progressing with NHS England to fund testicular tissue cryopreservation alongside ovarian tissue cryopreservation as part of a specialised service funding. At the present time, testicular tissue processing and storage is primarily funded by charitable donations and research funding. In Scotland, testicular tissue storage is funded by the NHS.

In regard to the funding of FP in adults and adolescents; although NICE recommend that FP in the form of gamete storage be offered to all with cancer, for 10 years or longer if fertility is at risk of significant impairment [40], access to NHS funds for this service is highly inconsistent. Decisions about eligibility criteria for NHS‐funded storage, and the duration of storage that will be paid for are made by Clinical Commissioning Groups and Health Boards apportioning NHS funds for healthcare in geographical areas in the UK. A patient’s access to FP is therefore largely determined by postcode. Typically, the storage of sperm is paid for the first 5 years, after which time, they may self‐fund the continued storage of their samples for around £350/year. There is a disconnect here with the centralised NHS funding of SSR procedures (Specialised Commissioning Team, 2016) and there is a call to improve access to and parity of NHS funding for FP services throughout the country [41].

## Conclusions

This paper highlights three different groups of patients who may require intervention from a surgeon to undergo FP prior to oncological treatment. The first, is the adult male who has been found to have azoospermia or unable to ejaculate and thus cryopreserve before their cancer treatment. The second, is the young prepubertal male in whom tissue containing SSCs can be retrieved and frozen for the future, with the anticipation that successful animal models will have been translated into human clinical application. The third group is the peri‐pubertal male in which there is some uncertainty as to the presence of sperm. By having a urologist and embryologist available in theatre, some young boys who would have gone straight for the SSCR procedure may be found to have sperm within seminiferous tubules. This result will completely change their future fertility potential.

The biggest obstruction to FP is the concern from the oncology teams that there is no time for a surgical retrieval if obtaining an initial semen sample is unsuccessful, in addition to concerns that a SSR may have complications that cause a further delay to treatment. The pressure to commence treatment as quickly as possible will often limit onward referrals to the urology team. Therefore, engaging with the cancer teams to encourage referrals with support for a small surgical procedure, which potentially can offer them paternity in the future, is the solution.

Additional factors that may also affect the possibility of offering FP in these patients include managing space on emergency surgical operating lists, anaesthetic concerns for often very unwell or paediatric patients, and liaising with embryologists and the stem cell cryopreservation teams to find urgent availability on a day suitable for all, which can all be extremely challenging. One option to improve the utilisation of FP services for patients, is to further involve urologists in the late effects’ clinics to build relationships within oncology/surgical teams. Creating multidisciplinary teams should improve opportunities to discuss and undergo FP.

In the present study, more than two‐thirds (68%) of males who had failed semen cryopreservation were able to have a successful SSR, giving themselves the potential for parenthood in the future. Emergency procedures were carried out within 7 days in 75% of men and all patients were able to commence further oncology treatment within 16 days (excluding the two deaths unrelated to the FP procedure). There was no delay to any cancer treatment secondary to surgical complications.

This paper reinforces the need for collaboration between oncologists and urologists to improve access to male FP services. It also demonstrates that these FP options can be undertaken without significant delay for cancer treatment.

## Disclosures of Interest

Lionel A. Micol’s institution receives consulting fees from Coloplast AG and Debiopharm International SA, payment for expert testimony from Dialectica Ltd. and meeting support from Debiopharm International. Lionel A. Micol has stock options in Cabinet d'Urologie and Andrologie, Dr Lionel Micol, Sàrl. Rod T. Mitchell received payment from Novo Nordisk as a guest speaker; is a MHRA Expert Working Group Member, and a Society for Reproduction and Fertility – Council Member.

**Abbreviations**
EEj: electroejaculation
FFPO: Future Fertility Programme Oxford
FP: fertility preservation
HTA: Human Tissue Authority
ICSI: intra‐cytoplasmic sperm injection
IVF: in vitro fertilisation
LH: luteinising hormone
MicroTESE: microscope‐assisted testicular sperm extraction
OCTB: Oxford Cell and Tissue Biobank
(onco‐)TESE: (oncological) testicular sperm extraction
OUHFT: Oxford University Hospitals NHS foundation Trust
PESA: percutaneous sperm aspiration
SSC(R): spermatogonial stem cell (retrieval)
SSR: surgical sperm retrieval
TESA: testicular sperm aspiration
UCLH: University College of London Hospital
